# Article of Significant Interest in This Issue

**DOI:** 10.1128/iai.00105-25

**Published:** 2025-03-11

## PROTEASE KEEPS SUPERANTIGENS IN BALANCE

Group A *Streptococcus* virulence is promoted by superantigen toxins that strongly activate T cells. Johnson et al. (e00405-24) show a critical dual role for a bacterial protease in the post-translational regulation of superantigens. Acting as a rheostat, it can degrade them to limit excessive activity and also amplify their activity through independent, complementary targeting of host cell signaling. These results show additional selection in the evolution of these toxins and new targets in the control of rheumatic fever and toxic shock syndrome.

